# How do we know that research ethics committees are really working? The neglected role of outcomes assessment in research ethics review

**DOI:** 10.1186/1472-6939-9-6

**Published:** 2008-03-28

**Authors:** Carl H Coleman, Marie-Charlotte Bouësseau

**Affiliations:** 1 Seton Hall Law School, Newark, New Jersey, USA; 2 Department of Ethics, Equity, Trade & Human Rights, World Health Organization, Geneva, Switzerland

## Abstract

**Background:**

Countries are increasingly devoting significant resources to creating or strengthening research ethics committees, but there has been insufficient attention to assessing whether these committees are actually improving the protection of human research participants.

**Discussion:**

Research ethics committees face numerous obstacles to achieving their goal of improving research participant protection. These include the inherently amorphous nature of ethics review, the tendency of regulatory systems to encourage a focus on form over substance, financial and resource constraints, and conflicts of interest. Auditing and accreditation programs can improve the quality of ethics review by encouraging the development of standardized policies and procedures, promoting a common base of knowledge, and enhancing the status of research ethics committees within their own institutions. However, these mechanisms focus largely on questions of structure and process and are therefore incapable of answering many critical questions about ethics committees' actual impact on research practices.

The first step in determining whether research ethics committees are achieving their intended function is to identify what prospective research participants and their communities hope to get out of the ethics review process. Answers to this question can help guide the development of effective outcomes assessment measures. It is also important to determine whether research ethics committees' guidance to investigators is actually being followed. Finally, the information developed through outcomes assessment must be disseminated to key decision-makers and incorporated into practice. This article offers concrete suggestions for achieving these goals.

**Conclusion:**

Outcomes assessment of research ethics committees should address the following questions: First, does research ethics committee review improve participants' understanding of the risks and potential benefits of studies? Second, does the process affect prospective participants' decisions about whether to participate in research? Third, does it change participants' subjective experiences in studies or their attitudes about research? Fourth, does it reduce the riskiness of research? Fifth, does it result in more research responsive to the local community's self-identified needs? Sixth, is research ethics committees' guidance to researchers actually being followed?

## Background

Many countries are investing significant resources in creating or strengthening "research ethics committees" (RECs) to review proposed research involving human participants, either within research institutions, as part of governmental agencies, or in the private sector. Implicit in these efforts is the assumption that REC review will result in research that better complies with applicable ethical principles. Yet, surprisingly little attention has been devoted to testing the empirical validity of this assumption. As a result, it is possible that countries are wasting scarce financial and human resources on processes that do not result in any real protections for research participants or their communities. In addition, without a system for evaluating RECs' actual impact, opportunities for remedying correctable problems with RECs are likely to be missed.

The importance of evaluating the effectiveness of RECs should be obvious. Yet, despite the emphasis on quality assessment in other areas of health care, "there has been near silence on the possibility of applying quality assessment techniques to ethics practices [1]." While the need for quality assessment in research ethics is beginning to receive greater attention [2], the focus has largely been on evaluating the quality of the deliberations that take place in RECs' meetings, as opposed to the impact of those deliberations on the research process itself.

Existing mechanisms for evaluating RECs are primarily limited to governmental or private auditing and accreditation programs. While both auditing and accreditation can make important contributions to the quality of research review systems, they are incapable of answering many critical questions about RECs' impact on research practices. Moreover, comprehensive auditing and accreditation programs require an investment of human and financial resources that is unfeasible for many low- and middle-income countries. In this article, we look beyond auditing and accreditation to consider other mechanisms for assessing and improving the quality of RECs' work.

While the focus of this article is RECs in low- and middle-income countries, the issues it addresses are relevant everywhere. In the United States, for example, an increasing chorus of critics has charged that the process of research ethics review imposes substantial costs for the research enterprise that exceed any benefits to research participants [3,4]. Determining whether the costs of ethics review are in fact justified requires a better understanding of the impact of ethics review on how research is actually performed.

## Discussion

### The Increasing Role of REC Review

REC review is a cornerstone of international guidelines on research with human participants. For example, the Council for International Organizations of Medical Sciences (CIOMS) states that "all proposals to conduct research involving human subjects must be submitted for review and approval to one or more independent ethical and scientific review committees [5]." Similar obligations appear in guidelines issued by the International Conference on Harmonization (ICH) [6], the Council of Europe [7], and UNESCO [8]. These guidelines require RECs to ensure that the risks of proposed studies are reasonable in relation to the anticipated benefits, that the investigators have adequate plans for obtaining participants' informed consent, and that other ethical issues, such as confidentiality and equitable participant selection, have been adequately addressed.

However, these international guidelines are not legally binding in countries that have not chosen to adopt them. Thus, whether REC review is required for any particular study depends on the requirements of national laws and policies. In the United States and many other wealthy countries, review by an REC is mandatory for most research involving human participants [9]. By contrast, in many low-income countries, there are no laws requiring REC oversight, or laws that exist are incomplete or under-enforced [10,11]. When research is conducted in collaboration with foreign research sponsors, some type of ethics review may be required by the laws of the sponsor's country, but such laws do not always require review by local RECs. For example, while the United States Food and Drug Administration (FDA) has extensive regulations governing REC review for clinical trials conducted pursuant to an Investigational New Drug Application (IND), those regulations do not apply to foreign trials not conducted pursuant to an IND. Instead, the FDA will accept data from non-IND foreign trials as long as the trial "conforms to the ethical principles contained in the Declaration of Helsinki [12]," which contains only very general provisions on ethical review.

In recent years, however, many low and middle-income countries have begun to pay greater attention to developing or strengthening RECs. For example, at the 2004 Ministerial Summit on Health Research in Mexico City, health officials from 58 countries called for national governments to adopt regulations providing for the "ethical oversight" of research [13]. In many African countries, governments have enacted, or are in the process of enacting, legislation requiring REC review of research involving human participants [14,15]. Even without a governmental mandate, many research institutions in resource-poor countries have created RECs on their own initiative, sometimes in collaboration with other countries [11] or with non-governmental organizations [16].

One reason for this increasing interest in RECs is that research sponsors are conducting more of their studies in low and middle-income countries, both because it is less expensive [17] and because it has become increasingly difficult to find a sufficient number of qualified participants in the sponsors' home countries [18]. In addition, a few highly-publicized controversies have led to greater attention to the potential for exploitation in the context of international collaborative research. For example, a lawsuit currently pending against the foreign sponsors of a Nigerian study of an anti-meningitis drug alleges that children in the control group were not given adequate medications, that parents were not told that effective treatment for meningitis was readily available outside of the study, and that documents claiming that the study had been approved by a Nigerian ethics review process were forged [19]. Publicity about cases like this has given sponsors a greater incentive to support the development of local RECs.

### Challenges for REC Review

For a country that lacks any research oversight system, creating a review process – *any *review process – is likely to have a positive impact. For example, requiring researchers to submit their protocols to RECs creates an incentive for researchers to actually have written protocols. Requiring them to document the informed consent process reduces the likelihood that individuals will be enrolled in studies without even being asked for consent. In other words, simply requiring prior approval to do research should help weed out the truly egregious cases of researcher misconduct.

Designing a system to evaluate the ethical acceptability of studies that pass this minimal screening function raises more challenging conceptual and practical difficulties. On the most basic level, the very concept of "ethics review" is inherently ambiguous, particularly in the critical area of risk-benefit assessment. Identifying the risks and potential benefits of research, and determining whether the balance between them is "reasonable," depend not only on scientific arguments but also on value judgments that usually have no clearly right or wrong resolution. In the absence of objective standards, RECs must rely on individuals' discretionary judgments, an approach that risks overemphasizing the personal values and biases of the individuals who happen to be serving on the committee [20]. In addition, the discretionary nature of risk-benefit assessment increases the potential for inconsistent decision-making, not only between different RECs but also within a single REC as it confronts similar issues from meeting to meeting. Of course, in a pluralistic society, absolute uniformity in ethical decision-making is neither realistic nor desirable. However, widespread inconsistency, particularly within a single REC, creates the impression that ethical standards are being applied in an arbitrary manner.

Some RECs deal with the amorphous nature of risk-benefit assessment by spending most of their time on detail-oriented questions that appear more susceptible to objective resolution, such as parsing the wording of informed consent forms [21]. Unfortunately, while rewriting consent forms is undoubtedly important in some situations, when it becomes the primary focus of ethics review larger ethical questions can easily become lost. Indeed, some critics charge that an obsessive focus on rewriting consent forms can actually undermine the protection of research participants, as it may simply result in longer and more confusing forms that participants will be less likely to understand [22].

On a more practical level, the effectiveness of REC review is often hampered by insufficient financial and human resources [15]. These limitations make it difficult to create committees with sufficient expertise and diversity, to provide funding for staff support, and to provide training for committee members. Ensuring the independence of RECs can also be a significant challenge. For example, in institutional-based RECs, committee members may be asked to vote on proposals submitted by colleagues who are personal friends, or by senior members of their department who control decisions about promotion and tenure.

The problem of independence is particularly acute for RECs in low and middle-income countries. Because such countries may depend on the financial or other benefits associated with foreign-sponsored research, RECs may be under explicit or implicit pressure not to reject research protocols or to insist on changes that might lead sponsors to take their studies elsewhere [23]. Adding to these problems is the fact that, in many resource-poor countries, RECs must carry out their work in the absence of a well-developed regulatory structure or a culture of compliance with administrative and procedural requirements.

### Existing Oversight Mechanisms for RECs

In many countries, REC oversight is the responsibility of national governmental agencies. In the United States, for example, the Food and Drug Administration (FDA) and the Office for Human Research Protections (OHRP) are responsible for overseeing most RECs in the country (where they are known as institutional review boards, or IRBs). These agencies conduct site visits of selected programs, either without cause or in response to a specific problem, and they also have less formal procedures for responding to individual complaints. OHRP's site visits include a review of 20–35 randomly-selected protocols and meeting minutes dating back one to four years [24].

Some countries require RECs to go through a formal process of governmental accreditation. For example, in New Zealand, the Health Research Council accredits research ethics committees. If a study proceeds without the approval of an accredited ethics committee, participants who suffer injuries may not be eligible for compensation from the country's no-fault compensation system [25]. Approval by an accredited committee is also necessary for researchers to obtain access to data held by the New Zealand Health Information Service database [26]. Accreditation usually involves a combination of self-assessment and external reviews, focusing on issues like committee membership, operating procedures, and the documentation of meetings [27].

There are also voluntary accreditation programs for RECs. The largest of these is run by the Association for the Accreditation of Human Research Protection Programs (AAHRPP) [28]. Obtaining AAHRPP accreditation is an intensive process that usually takes 12–18 months, including both document reviews and a 2–4 day site visit during which dozens of persons involved in all aspects of the research program are individually interviewed. In order to be accredited, programs must demonstrate not only that they are in compliance with all applicable regulatory requirements, but also that they have developed guidelines for addressing certain issues not expressly covered by the regulations (e.g., standards governing the participation of decisionally incapacitated persons in research). While most of the programs that have received AAHRPP accreditation are located in the United States, AAHRPP has also accredited programs in Canada, Singapore, and South Korea.

In addition to AAHRPP, the Strategic Initiative for Developing Capacity in Ethical Review (SIDCER), a network of local and regional ethics organizations working with several United Nations organizations, has established a voluntary "recognition" program for RECs. The program offers recognition to RECs that demonstrate that they "(1) have a structure and composition appropriate to the amount and nature of research being conducted; (2) have appropriate management and operational procedures; (3) review protocols in a timely fashion according to established procedure; (4) adequately and effectively communicate decisions to investigators; and (5) have appropriate practices regarding documentation and archiving [29]." The SIDCER program includes educational components designed to support RECs' progress toward recognition. Committees from China, Philippines, South Korea, Thailand and Taiwan have already been recognized through this process.

Finally, some mechanisms also exist for evaluating the qualifications of individual REC members. For example, a non-profit organization in the United States called Public Responsibility in Medicine and Research (PRIMR) offers a certification program for IRB members and staff, which "evaluates and validates individuals' knowledge of ethical principles, historical events, regulatory requirements, and operational and functional issues relating to IRBs and other human subjects protection programs [30]." Persons who pass the certification test are authorized to include the acronym CIP ("certified IRB professional") in their professional titles.

All of these mechanisms can make important contributions to the quality of the ethics review process. Auditing and accreditation programs encourage RECs to develop standardized policies and procedures, which helps promote the consistent application of ethical principles. They also provide a means for checking whether RECs are actually adhering to the policies and procedures they claim to be following. Private accreditation programs have the added advantage of encouraging RECs to develop policies and procedures for issues that are insufficiently addressed at the regulatory level. Certification programs for REC members can complement the accreditation process by promoting a common base of knowledge about applicable ethical and regulatory principles. Moreover, both accreditation and certification are likely to enhance the status of RECs within their own institutions, which may make it easier for RECs to gain necessary institutional resources.

However, these mechanisms also have inherent limitations. Most significantly, they focus primarily on questions about RECs' structure and process, such as how committees are constituted, whether their standard operating procedures are complete, and whether the process of protocol review is adequately documented [31]. One danger with this focus is that it may exacerbate RECs' tendency to emphasize form over substance. A recent study of OHRP enforcement activities highlights this problem; it found that "the agency continues to nitpick consent forms, depends upon (and demands) extensive documentation of compliance activities, and finds the remedy for most problems to be 'more' – review of studies, internal monitoring procedures, education, forms." The result of this focus, the authors conclude, is "a culture of red tape rather than a culture of ethics [24]."

A larger concern with an exclusive focus on structure and process is that it is incapable of answering the bottom-line question: whether REC review actually protects the rights and interests of research participants and their communities. For example, the fact that an REC has documented that it has considered a protocol's risks and potential benefits does not mean that it has done a good job identifying or weighing these factors. Nor does it show that studies that are approved by the committee have more favorable risk-benefit profiles than those that are turned down. Similarly, the fact that an REC has concluded that a consent form contains all relevant information does not mean that prospective participants who read the form will actually understand or absorb the information, or that it will have any appreciable impact on their decision-making process. In short, all that auditing and accreditation programs tell us is whether RECs are carrying out the specific tasks that have been assigned to them. While this is certainly an important question, even an affirmative answer does not prove that an REC system "works."

### Integrating Quality Assurance Principles into REC Assessment

Principles developed through decades of experience with quality assurance and improvement in other areas of health care can guide the development of effective oversight programs for RECs. The most basic of these principles is that, before the quality of any program or service can be evaluated, the relevant elements of quality must be identified with precision. Quality is a multifaceted concept, including factors like the technical competence with which an activity is performed, the impact of the activity on morbidity and mortality, and the activity's cost effectiveness. Choosing which of these goals to emphasize "requires a commitment to finding out what patients and the community need, want, and expect from the health service [32]."

Thus, the first step in developing a comprehensive quality assurance program for RECs should be to identify what prospective research participants and their communities hope to get out of the ethics review process. For example, is the concern that people are being misled into enrolling in studies in which they would have refused to participate had they known what they were getting into? If so, it would be useful to know whether REC review has an impact on the number of people who go through the informed consent process and then decide not to participate. Alternatively, the goal might be to increase the extent to which participants feel respected in the research process, regardless of whether they end up making different decisions about participating [33]. In that case, we should try to find out whether REC review actually affects participants' subjective experiences in studies or their attitudes about research. Both of these questions, of course, depend on first determining whether prospective participants understand the information that has been presented to them in the consent process. While some research related to this question has already been conducted [34], assessing participants' understanding has not yet been systematically integrated into the process of REC oversight.

Similar questions can be raised about the process of risk-benefit assessment. For example, is REC review considered important because of a concern that research is generally "too risky"? If so, we should look at whether adopting an REC process actually affects the riskiness of research – perhaps by investigating whether REC review has an impact on the incidence of adverse events. Or is the concern that, without RECs, research might not address the health needs of the local community? In that case, we might want to see whether studies approved by an REC are in fact consistent with the local community's needs.

These broad outcome-oriented questions will not always be easy to answer. Outcomes assessment is one of the trickiest areas of quality assurance; numerous confounding variables can undermine the validity of simple before-and-after comparisons. For example, in many health care programs, outcome measurements such as mortality rates can be useful indicators of the quality of services, but they may also reflect "differences in the resources available, the risk factors of the patient group, data accuracy, and chance alone [35]." Likewise, an increase in adverse events following the creation of an REC does not necessarily mean that the REC is a failure; it may instead reflect a shift towards studies related to more serious conditions, where the greater potential benefits justify a higher degree of risk. Developing methodologically sound measures of assessing REC outcomes is an area ripe for further research.

Another important dimension of quality assurance that existing oversight efforts do not incorporate is the basic question of whether RECs' guidance to researchers is actually being followed. For example, do researchers really utilize the informed consent processes described in their protocols, or do they simply hand prospective participants a consent form and ask them to sign it? RECs could adopt a variety of relatively simple methods to generate information relevant to this question, such as soliciting feedback from prospective participants through questionnaires or suggestion boxes. Or, borrowing a practice used in other health care settings, they could use actors to play the role of prospective research participants, in order to evaluate how people are treated when they go through the informed consent process [32]. While these measures would require some additional resources, they are far less expensive than many other methods of quality assurance – for example, going through an 18-month process of obtaining accreditation.

Finally, and most importantly, a "monitoring system is not an end in itself [36];" the information generated through an assessment process must be used to stimulate improvements in practice. Doing this requires a commitment to a process of continuous quality improvement, in which information from the assessment process is disseminated to key decision-makers and incorporated into practice. Thus, an REC might ask researchers involved in approved studies to report back about the most common questions asked by prospective participants during the informed consent process, and then use this information to change the way they evaluate informed consent forms in the future. Global or regional meetings of RECs, as well as online discussion forums, can provide valuable opportunities for sharing information and identifying and promoting best practices. In addition to disseminating information among REC members, it is important to share findings about REC practices with external audiences like administrative authorities and community leaders.

## Conclusion

Low and middle-incomes countries are increasingly demonstrating their capacity to put into practice concrete mechanisms for enforcing ethical requirements. International cooperation has been a key factor in this progress. However, it is not clear whether these systems have led to substantial improvements in the way that research is actually conducted. It is time to look beyond the basic question of whether RECs are complying with existing standards to the larger question of whether compliance with these standards is having the desired results.

## Competing interests

The author(s) declare that they have no competing interests.

## Authors' contributions

CHC and MCB jointly developed the ideas presented in this article. CHC wrote the first draft, and both authors jointly revised the draft. Both authors read and approved the final manuscript.

## Pre-publication history

The pre-publication history for this paper can be accessed here:


